# Spectra Reconstruction for Human Facial Color from *RGB* Images via Clusters in *3D* Uniform CIELab* and Its Subordinate Color Space

**DOI:** 10.3390/s23020810

**Published:** 2023-01-10

**Authors:** Suixian Li, Kaida Xiao, Pingqi Li

**Affiliations:** 1 Flying College, Binzhou University, Binzhou 256600, China; 2 School of Design, University of Leeds, Leeds LS2 9JT, UK; 3 School of Informatic, University of Edinburg, Edinburgh EH8 9YL, UK

**Keywords:** human facial color, spectra reconstruction, *RGB* images, high fidelity color reproduction, *PCA* (Principal Component Analysis), polynomial regression, subordinate color subspace

## Abstract

Previous research has demonstrated the potential to reconstruct human facial skin spectra based on the responses of *RGB* cameras to achieve high-fidelity color reproduction of human facial skin in various industrial applications. Nonetheless, the level of precision is still expected to improve. Inspired by the asymmetricity of human facial skin color in the CIELab* color space, we propose a practical framework, *HPCAPR*, for skin facial reflectance reconstruction based on calibrated datasets which reconstruct the facial spectra in subsets derived from clustering techniques in several spectrometric and colorimetric spaces, i.e., the spectral reflectance space, Principal Component (*PC*) space, CIELab*, and its three *2D* subordinate color spaces, La*, Lb*, and ab*. The spectra reconstruction algorithm is optimized by combining state-of-art algorithms and thoroughly scanning the parameters. The results show that the hybrid of *PCA* and *RGB* polynomial regression algorithm with *3PCs plus 1st-order* polynomial extension gives the best results. The performance can be improved substantially by operating the spectral reconstruction framework within the subset classified in the La* color subspace. Comparing with not conducting the clustering technique, it attains values of 25.2% and 57.1% for the median and maximum errors for the best cluster, respectively; for the worst, the maximum error was reduced by 42.2%.

## 1. Introduction

Interest in spectral reflectance, as one of the optical properties of human facial skin, has been growing in various industrial contexts, such as facial detection in transportation [1], skin pigmentation prediction for the cosmetic industry [2,3], skin color modeling in computer graphics [4,5], skin color measurement for the diagnosis of cutaneous diseases, and skin color matching for body and facial prostheses [6,7]. Once we have evaluated the spectral reflectance of skin, its color under any known illumination can be computed according to CIE Colorimetry [8]. Furthermore, skin spectra can also be used to predict skin chromophores, providing an opportunity to extract important health-related information [9,10,11,12].

Among the various methods that measure skin color or its spectral reflectance [13,14,15,16,17,18,19,20,21,22,23], optical hyperspectral imaging is non-invasive with high efficiency, because the spectral reflectance of every pixel of the imaging target can be reconstructed in a single imaging cycle [17,18,19]. For example, Acquis S. L. acquires tissue optical properties via a hyperspectral imaging system with the aid of a light transportation model [19]. However, hyperspectral or multispectral imagers used for skin spectral imaging are considerably bulkier and more expensive than consumer cameras capturing *RGB* images [20,21,22,23,24,25].

In this field of skin spectra reconstruction from RGB facial images, there are two main objectives: bio-information extraction and color reproduction. Bio-information extraction imaging is based on the physical and physiological characteristics of human skin [19,20]. Nishidate et al. instantaneously estimated the levels of melanin and hemoglobin based spectral reflectance data from an *RGB* image [20]. As for the high-fidelity color reproduction of human facial skin color, the goal is to reconstruct the spectral reflectance; this would make it possible to precisely reproduce the color by softcopies or hardcopies under arbitrary illumination conditions, according to the von Kreis color consistency principle [26].

In this article, we focus on reconstructing the spectral reflectance of human facial skin from *RGB* images and propose a novel framework for the calibration of the system. Our approach adopts hybrid *1st order RGB* polynomial regression and a *3PCs PCA* imaging model to predict the spectral reflectance from the camera response in *RGB* images via a priori training data classified in the 2D La* color subspace. The training data comprise measured reflectance spectra and the corresponding RGB triplets of the facial color of the subject under specific illumination and other consistency imaging parameters. In this way, the reflectance spectra can be precisely reconstructed from the RGB triplets acquired using the same imaging parameters.

The methodology and superiority of the proposed method is discussed in the following sections. In Section 2, we review related works. In Section 3, we introduce the hybrid *CPA* plus *PR* framework (*HPCAPR*) for optimal transformation based on the *RGB* response of a consumer camera to the reflectance spectra of human facial colors. The experiment design is described in Section 4. In Section 5, the experimental results are detailed, confirming the superior performance of the proposed HPCAPR method. Two sets of HPCAPR configurations are investigated according to a logical investigation carried out by our team, spanning several years. In Section 5.1, we provide data justifying our claim that the *3PCs plus 1st-Order* algorithm is the best existing algorithm. In Section 5.2 and Section 5.3, the 2D La* subordinate color space and the *cosine distance* are confirmed to be the optimal choices. To the best our knowledge, our study is the first to conduct reflectance reconstruction for human skin in the 2D La* color space applying the described configuration in the HPCAPR method.

## 2. Related Works

To properly contextualize the framework proposed in this article, a brief review of related works is included in this section.

Based on the measured reflectance spectra, the corresponding colors in *RGB* images of the facial skin of several subjects, *PCA* [27,28] method, and *PR* [29] technique, Imai transformed human skin color images captured by *HDTV RGB* cameras to color printing chips and colors displayed them on a CRT in the mid-1990s [21]. Xiao et al. transformed camera *RGB* directly to skin reflectance spectra using a *RGB* polynomial regression and PCA projection approach (DPRPCA) [22]. R. He et al. transformed raw *RGB* to spectra by first order polynomial regression [23,24] using different calibration datasets. Based on Xiao’s method [22], Ma L. et al. transformed *RGB* to spectra by second order polynomial regression and *3PCs* projection with regulated denoise items [25].

The motivation of the above heuristic methods is similar, namely, *high-fidelity color reproduction*. Although all of these methods are optimized on the bases of real skin reflectance spectra and camera *RGB* response (viz. spectral datasets), the algorithms they use are substantially different. The relevant algorithms, along with the calibration methods, are summarized in Table 1. We can see that the PCA and PR methods are mainstream approaches for skin color reproduction. Nevertheless, due to the use of different training sets in calibrating the spectra reconstruction, the order of the regression polynomial and the number of *PCs* may vary. Except for [25], which described the use of a denoising strategy [25], all presented approaches allowed us to explore adaptive methods and their effectiveness, rather than focusing on trivial computational residual errors.

## 3. Method

In Section 3.1, we present the algorithms used in the approaches described in Section 2. Then, in Section 3.2, we introduce the hybrid *CPA* and *PR* spectral reconstruction framework (*HPCAPR*) for optimizing the transformation from the *RGB* response of a consumer camera to the reflectance spectra of human facial colors. In Section 3.3, we propose a methodology to reconstruct the reflectance of human skin via subordinate clusters. The motivation for this approach is twofold. We will disclose the nature of the *HPCAPR* framework and the idea of reconstructing human skin reflectance in clusters in terms of a subordinate color space in the interests of drawing comparisons between the proposed and the aforementioned algorithms.

### 3.1. HPCAPR Spectral Reconstruction Framework

#### 3.1.1. General Two-Step Algorithm

Generally, reflectance reconstruction involves two steps, i.e., spectral characterization and reconstruction, on the condition that the illuminant and spectral sensitivity of the camera sensor are unknown.

In the characterization step, the spectral characteristics of the imaging system are specified by the transform matrix (also called projection) **M** using response matrix $C_{Tr}$ and the corresponding measured reflectance $R_{Tr}$ in the training set (cf. Equation (1)).

(1) $$C_{Tr}=MR_{Tr}$$

Under the same imaging conditions as those used during training, reflectance $R_{Re}$ could be accurately reconstructed from the matrix of the RGB triplets from images $C_{Re}$ in the second step if the projection matrix M was optimized. See Equation (2), where $M^{+}$ is the pseudoinverse matrix of M**.**

(2) $${R_{Re}=M}^{+}C_{Re}$$

For the sake of the optimization of matrix M, a second verification step is used for evaluation via the perceptual color difference metrics, for instance, ΔE*ab (CIEDE Lab*) and/or the spectral difference *RMSE* (Root Mean Square Error) between the reflectance spectra in the verification set and its corresponding reconstructed ones. In Equation (3), $R_{Veri}$ is the matrix of reflectance spectra in the verification set, and $C_{Veri}$ is the matrix of the corresponding *RGB* triplets of camera response.

(3) $${R_{Veri}=M}^{+}C_{Veri}$$

For more clarity, the dimensionalities of the matrices in the above equations are explicated as follows. The columns of matrices $C_{Tr}$ and $C_{Re}$ are equal to the spectral channels of camera *m*; rows of $R_{Tr}$ and $R_{Re}$ are equal to the dimension of reflectance *n*. Therefore, the dimensions of transform matrix M are equal to m×n, and those of $M^{+}$ are n×m. The rest of the dimensionalities comprising the rows of $C_{Tr}$, the columns of $R_{Tr}$, and the correspondents of $C_{Re}$ and $R_{Tr}\;are$ equal to the number of samples in the training set and the number of samples to be reconstructed, respectively.

The reflectance reconstruction algorithm aims to transform the responses of the camera, *m =* 3 dimensional RGB triplets, to spectral reflectance with, supposing the same dataset is used, *n =* 31 dimensions. Therefore, the direct inverse transformation is always ill-conditioned. As can be seen, we call this method *direct inversion*. In principle, reducing the ill condition of the transform matrix is crucial to improve the accuracy of reflectance reconstruction. Therefore, intuitive but reasonable strategies to overcome this obstacle involve either extending the dimension of camera response RGB triplets from 3 to a greater number or reducing the dimension of reflectance from 31 to a lower number. This is the fundamental mathematical reason for the use of polynomial regression (*PR*) and the *PCA* method, which are described in Section 3.1.2 and Section 3.1.3.

It is worth noting that pursuing the orthogonality of transform matrix M is another way to increase the precision of the reflectance estimation. At the same time, it is also an ideal theoretical criterion for selecting the parameters for spectral imaging. As a consequence, the authors have put forward many spectral reconstruction algorithms based on the filter selection, training set selection, and noise reduction methodologies, among others [21,25,28,29,30,31,32,33,34,35,36]. Detailed discussion about this topic is outside of the scope of this article.

#### 3.1.2. Polynomial Dimensional Extension

In this case, matrix **M** in Equation (1) is derived from the polynomial extension of the *RGB* responses of the camera, that is to say, the columns of matrix $C_{Tr}$ are the vectors of polynomial extension of the *RGB* triplets of the training set samples. In this research, the dimension of reflectance is 31. For the sake of overcoming the ill condition of matrix **M**, the possible order of the polynomial can be 1, 2, 3, 4, and the numbers of the corresponding items of the polynomial can be 4, 10, 20, and 35 respectively. The components of RGB polynomial extension are:

**A**_1_ = [*1 R G B*]; (4)

**A**_2_ = [*RR GG BB RG RB GB*]; (5)

**A**_3_ = [*RRR GGG BBB RRG RRB GGR GGB BBR BBG RGB*]; (6)

**A**_4_ = [*RRRR GGGG BBBB RRRG RRRB GGGR GGG*

*BBBR BBBG RRGG RRBB GGBB RRGB GGRB BBRG*]. (7)

where the *R*, *G*, and *B* in the square brackets are the values of the pixels in the *RGB* images; each item separated by a space is an element of a row vector. In each item, a group combining *R*, *G*, or *B* indicates the multiple of these values. As above, the order of the polynomial equals the highest number of multipliers in an element. Superscript *t* denotes the transposition of a matrix, and **P*_rgb_***_i_ (*i* = 1, 2,..., *j*) is the column vector of the *i*th order polynomial.

**P***_rgb j_* = [**A**_1_
**A**_2_
**… A***_j_*]*^t^*, *j* ≤ 4. (8)

where the arrangement of the elements of the corresponding vector follows the expansion convention of a row vector in Matlab, e.g., **P***_rgb_*_1_ = [1 *R G B*]*^t^*, **P***_rgb_*_2_ = [1 *R G B RR GG BB RG RB GB* ]*^t^*, and so on. Regarding polynomial extension, the number of items in the corresponding *1^st^~4^th^* order of polynomials would be 4, 10, 20, and 35.

Therefore, the spectral reconstruction process can be expressed as:

(9) $${P_{rgbi}}_{Tr}={M_{PR}R}_{Tr};$$

(10) $$R_{Re}={{M_{PR}^{+}P}_{rgbi}}_{Re}$$

(11) $$R_{veri}={{M_{PR}^{+}P}_{rgbi}}_{Veri}$$

where $M_{PR}$ is the transform matrix from the reflectance of training set $R_{Tr}$ to the *i*th order polynomial extension of the *RGB* triplets of training set ${P_{rgbi}}_{Tr}$; $M_{PR}^{+}$ is the Moore–Penrose pseudo-inverse of matrix $M_{PR}$; subscripts Re,Tr and Veri denote the items related to the reconstructed ones, the calibration training set, and the verification set, respectively; and subscript PR of the transform matrix corresponds to the *polynomial regression (PR)* method.

#### 3.1.3. PCA Dimension Reduction

Rather than extending the dimension of the response of the camera from three items to a higher number (as in the polynomial regression method), the *PCA* method projects reflectance to a lower dimensional spectral space using only a few eigenvectors of the reflectance:

(12) $$R_{Tr}=U_{j}^{t}\alpha ;$$

where **U** is the matrix of *PCs*, or the *principal component matrix*; subscript *j* denotes the number of the first *j* of the most significant *PCs*; superscript *t* denotes the transposition of the matrix; each row vector of $U_{j}^{t}$ is an eigenvector of the *PC source* referred to in Table 1; and α is score vector in the columns of the training set. It can be deduced that the number of elements in a column vector in matrix α should be equal to *j*. In this way, the 31-dimensional of reflectance is reduced to *j*. Then, the spectral reconstruction process parallel to Equations (9)–(11) would be:

(13) $$C_{Tr}=M_{PCA}R_{Tr}=M_{PCA}U_{j}^{t}\alpha ;$$

(14) $$R_{Re}=U_{j}^{t}\beta_{Re}=M_{PCA}^{+}C_{Re};$$

(15) $$R_{Veri}=U_{j}^{t}\beta_{Veri}{=M}^{+}C_{Veri};$$

where $\beta_{Re}$ is the score matrix corresponding to the reflectance to be reconstructed, similar to $\beta_{Veri}$; subscript PCA, of the transform matrix corresponds to the *PCA* method. From Equations (13)~(15), it follows that:

(16) $$R_{Veri}=U_{j}^{t}{{\alpha C_{Tr}^{t}(C}_{Tr}C_{Tr}^{t})}^{-1}C_{Veri}.$$

The essence of *PCA* is the least square approach using a low-rank approximation of the data matrix. The lower rank matrix is supposed to be known a priori, but it can be estimated using singular linear prediction matrix **U**.

### 3.2. Hybrid Spectral Reconstruction Framework (HPCAPR)

#### 3.2.1. Combining PR and PCA

A logical approach is to combine the two methods, *PR* and *PCA*, as this would optimize the spectral reconstruction processing on both sides. Therefore, the hybrid method could be expressed similarly to Equations (9)−(11) and Equations (13)−(15):

(17) $$C_{Tr}=M_{Hbr}U_{j}^{t}\alpha ={P_{rgbi}}_{Tr};$$

(18) $$R_{Re}=U_{j}^{t}\beta_{Re}={{M_{Hbr}^{+}P}_{rgbi}}_{Re};$$

(19) $$R_{Veri}=U_{j}^{t}\beta_{Veri}=M_{Hbr}^{+}{P_{rgbi}}_{Veri}$$

where the subscript *Hbr* of the transform matrix corresponds to the hybrid *PCA and PR combining* method. Deriving from Equations (13)–(15), we obtain:

(20) $$R_{Veri}=U_{j}^{t}\alpha {P_{rgbi}}_{Tr}({{P_{rgbi}}_{Tr}{{P_{rgbi}}_{Tr}}^{t})}^{-1}{P_{rgbi}}_{Veri}.$$

Note that the two parameters in Equation (20), i.e., the number of polynomial orders *i* and the number of *PCs j*, will be determined by *parameter scanning* in the following Section 4.2.

#### 3.2.2. Spectra Reconstruction in Clusters Classified in a Subordinate Color Space

**Figure 1:**
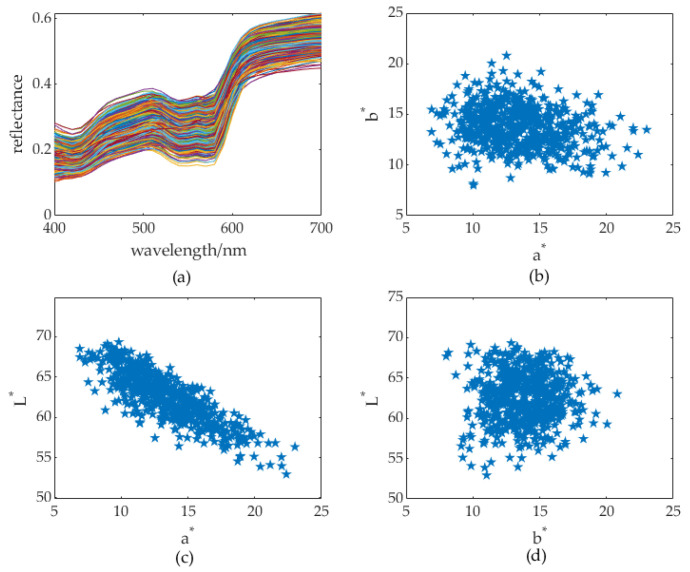
Asymmetry distribution of a skin color assembly in spectral and color space. (**a**) Reflectance spectra space, (**b**) 2D a*b* color subspace, (**c**) 2D L*a* color subspace, (**d**) (C) 2D L*b* color subspace. Note that the different colors of the curves in (**a**) are randomly generated by plotting tools.

Minimizing the square error to estimate unknown variables from a priori knowledge is a general approach for spectral reconstruction. In practice, a priori knowledge is abstract in the training set. An ideal training set should be comprised of the most representative samples that span the space of the assembly, such that those that are not in the training set can be reconstructed accurately. Previous research has selected training sets based on various criteria (see Table 1); however, we adopt a different method to the select training set from clusters in a *3D* uniform CIELab* color space and its subspace. Our method is inspired by the substantial asymmetricity of human skin colors in the uniform color space CIELab* (see, Figure 1). Details of the acquisition of the displayed dataset are provided in Section 4.1. From Figure 1, we can see that the skin colors in the La* plane are much more asymmetric than those in the a*b* and Lb* planes (hereafter called the *2D* color subspace).

The heuristic method contains two steps. The first is to classify the skin dataset assembly into clusters using the k-means algorithm [37]. This can be performed using the *Matlab* function *kmeans*. Given the number of the clusters to be classified, i.e., *k*, the *k-means* function partitions ensemble data *S* that contains *N* samples into *k* clusters (*S*_1_, *S*_2_,*…*, *S*), minimizing the sum of point-to-centroid distances *D(X_i_*, *X_c_*,*_m_)*, summed over all *k* clusters:

(21) $$S_{m}=\{X_{i}|min[\sum_{i=1}^{i=N}D(X_{i},X_{c,m})]\},\;m\in \left\{1,2,..,k\right\}$$

Several distances *D(X_i_*, *X_c_*,*_m_)* could be used:

(1) ‘sqeuclidean’ distance. Each centroid is the mean of the points in that cluster.

(22) $$D_{Sqeuc}\left(X_{i},X_{c,m}\right)=\left(X_{i}-X_{c,m}\right){\left(X_{i}-X_{c,m}\right)}^{t}$$

(2) ‘cityblock’ distance. Each centroid is the component-wise median of the points in that cluster.

(23) $$D_{CB}\left(X_{i},X_{c,m}\right)=\sum_{i=1}^{p}\left|X_{i}-X_{c,m}\right|$$

(3) ‘cosine’ distance. Each centroid is the mean of the points in that cluster after normalizing those points to Euclidean length.

(24) $$D_{cos}\left(X_{i},X_{c,m}\right)=1-\frac{X_{i}X_{c,m}^{t}}{\sqrt{\left(X_{i}X_{i}^{t}\right)\left(X_{c,m}X_{c,m}^{t}\right)}}$$

**Figure 2:**
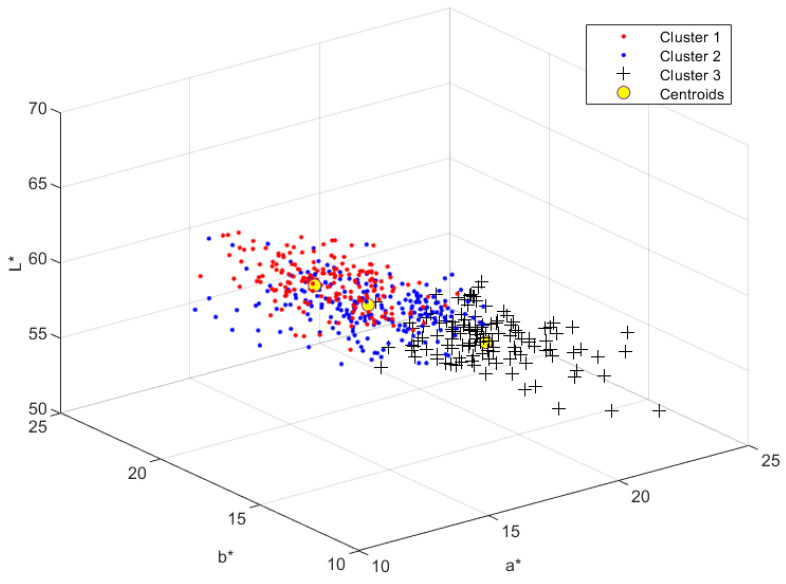
Clusters classified by *kmeans* (*k = 3*) with ‘sqeuclidean’ distance in the CIELab* color space.

Figure 2 shows an example of skin colors classified by kmeans (*k* = 3) with ‘sqeuclidean’ distance in the CIELab* color space.

The second step applies the spectral reconstruction framework *HPCAPR* within subsets *S_m_*. In Section 5, we show that the cluster obtained using the ‘*cosine distance*’ in the 2D La* color space goes beyond the other clusters.

It is worth noting that the notations in this section are independent of those used in other sections for clarity. For instance, whether $X_{i}$ denotes color coordinates or reflectance vectors depends on which space of the clustering is to be transformed.

### 3.3. Results Evaluation

To evaluate the quality of the spectral reconstruction, many indices can be used [27]. Generally, the indices are categorized into spectrometric and colorimetric types. We chose the two most widely used metrics, DELab* (${∆E}_{ab*}$) and *RMSE* (Equations (25) and (26)). DELab* is the color difference based on CIE Colorimetry, assuming a standard observer (CIE 1931 standard 2 deg observer) and a given illumination. *RMSE* considers only the spectral difference between the *n*-dimensional predicted $\hat{R}$ and measured spectral reflectance R. It is not affected by the illumination.

(25) $${∆E}_{{ab}^{*}}=\sqrt{{\left({\hat{L}}^{*}-L^{*}\right)}^{2}+{\left({\hat{a}}^{*}-a^{*}\right)}^{2}+{\left({\hat{b}}^{*}-b^{*}\right)}^{2}}$$

(26) $$RMSE=\sqrt{\frac{\left\|\hat{R}-R\right\|}{n}}$$

## 4. Experiment Design

### 4.1. Datasets

Two skin color datasets containing 514 and 608 effective samples, respectively, are used in this research. In the former, 282 samples were measured on Chinese and 232 on Caucasian subjects. The samples were collected with the same protocol as that in [22]. For each subject, the skin color measurements were obtained from three body areas: forehead, cheeks, and neck. A Konica Minolta CM-700d spectrophotometer was used to measure reflectance. Its viewing geometry is d/8 (diffuse illumination, 8-degree viewing) and the aperture size was set to 3 mm. The output spectral reflectance data were in the range of 400 nm to 700 nm, sampled at intervals of 10 nm. Facial images were captured using a Nikon D7000 DSLR camera with subjects sitting in a Verivide facial image viewing cabinet with diffused D65 lighting. Each facial image was saved as a camera *RGB* image. The *RGB* values corresponding to the former measured position were averaged from the area in the facial images with a diameter of approximately the same size as the aperture of the spectrophotometer. Except for the numbers of samples, the two datasets had no discernable differences.

**Figure 3:**
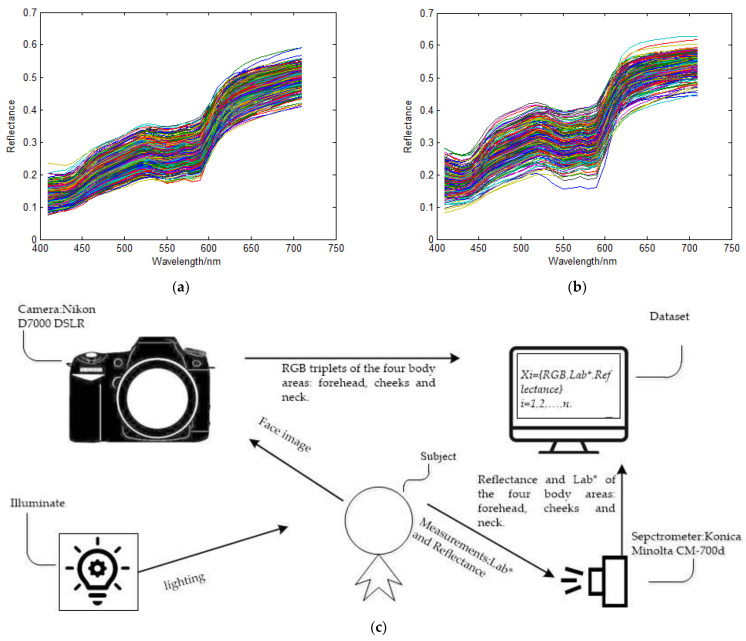
Reflectance display and dataset acquisition protocol. (**a**) Chinese, (**b**) Caucasian; (**c**) Schematic diagram of dataset acquisition and structure. Note that the different colors of the curves in (**a**,**b**) are randomly generated by plotting tools.

Figure 3 displays reflectance curves of the skin samples of subjects of two ethnicities in the dataset based on 514 samples and the schematic of dataset acquisition protocol. The dataset displayed in Figure 1 is the one containing 608 samples, which was applied as described below. In Figure 3c, we illustrate the data acquisition framework and the structure of the dataset. As indicated, a record of a sample of the dataset contains one record of the RGB response of the subject, one corresponding reflectance, and one color-coordinate Lab*. Among them, the former was acquired by averaging the RGBs of the pixels of the four areas on subjects’ forehead, cheeks, and neck from the RGB image taken by the camera. The size and positions of the areas correspond to the aperture size of the spectrometer and the measured position on the subject’s skin due to the immediate touched measure mode of the spectrometer. The latter, namely, the reflectance spectra and the Lab*, were measured from the same areas where the RGB responses of the camera were recorded for each subject.

In this article, the samples of the dataset have specific usages in terms of different computational missions. If one is familiar with the related research, such as that listed in Table 1 [21,22,23,24,25], the nature of the usage of the dataset may be known. In that case, the readers should feel free to skip to the next section.

In the characterization step of the reflectance reconstruction computation, the RGBs and the reflectance spectra of the training set were used as input data. We obtained the output, namely, the transform matrix, from RGB to reflectance. In the verification step, the RGB of the verification set (testing set) were used as input. With the help of the intermediate transform matrix, we obtained the output, namely, reconstructed reflectance $\hat{R}$. At the same time, the corresponding errors were calculated for evaluating the performance of the spectra reconstruction.

In the clustering computation, the choice of the input variant was made based on the spaces conducting the clustering operation; for example, the Lab* records of the dataset were used as input when clustering in Lab* color space. One may ask the following question: If we have the RGB response of the camera and without the Lab* and the reflectance, because these are not contained in the dataset, which cluster of the *RGB* belongs in the Lab* space? The answer is explicit. It is easy to map the *RGB* to Lab* with the aid of the known dataset. Details of the mapping method are beyond of the scope of this article.

Here, we take Equation (20) as an example, due to the fact that it contains both steps, i.e., characterization and verification; similar to the methods described above, the *RGBs* as well as the *PCs* of the reflectance samples all seems to be inputs. Therefore, it is hard to tell which records are the inputs of this calculation. However, if we recall the above derivation, we find that the training set reflectance spectra calculated using the $U_{j}^{t}\alpha$, the *RGBs* of the training set and verification set arranged in ${P_{rgbi}}_{Tr}$ and ${P_{rgbi}}_{Veri}$ are all input variants. If we calculate errors using Equations (25) and (26), the Lab* and the reflectance records of the verification set can be used as the inputs.

### 4.2. System Optimalization via Parameter Scanning

**Figure 4:**
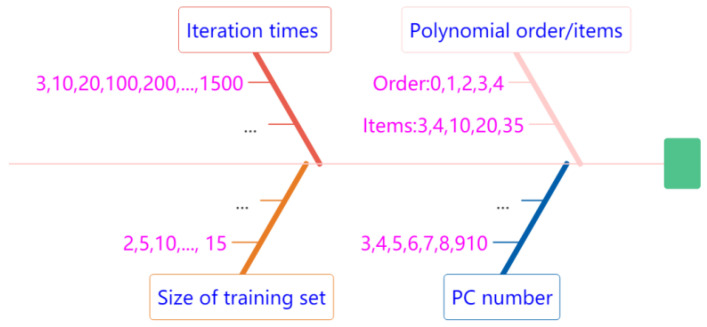
Schematic of the process of scanning for optimal parameters within reasonable ranges.

Four parameters are required for optimization. Figure 4 is a schematic of the scanning process used to obtain the optimal parameters.

The orders of polynomial *i* and the number of *PCs j* in Equation (20) follow the combinations from the corresponding sets. The order of *RGB* polynomial extensions *i* ∈ {0,1,2,3,4} correspond to items {3,4,10,20,35}. Note that the *0-order* polynomial contains just three items, that is, the alternative expression is **A_0_** = [*R G B*], similar to Equation (4). Because other researchers concluded that skin color could be reproduced sufficiently with *3PCs* [21,22,25], and more *PCs* contain more spectral information from the training set, we decided that a possible optimal number of *PCs* could be *j* ∈ {3,4,5,6,7,8,9,10}. Consequently, the number of combinations of the two parameters is 40, as shown in Table 2.

### 4.3. Further Scanning for Optimal Clusters

In order to find the minimum size of the training set, we started with *two* samples with reasonable step-sizes in ascending sequence and stopped at a certain number when the results were acceptable. In the second half of the experiment, we selected the training samples randomly to determine the optimal iteration times due to the fluctuation of the result of a single operation. Then, the other two parameters, i.e., the size of the training sets and the iteration times, were taken from the numbers listed in Figure 4.

The motivation here was to explore the optimal subspace classification using cluster techniques for skin reflectance reconstruction. The subspace could be either in the spectral reflectance domain, which comprises the direct spectrum domain and the domain projecting to *PCA*, or the subspace classified in CIELAB* and its derived *2D* subordinate color space a*b*, La*, Lb*.

## 5. Results and Discussion

### 5.1. Optimal Parameters for the Hybrid Algorithm

#### 5.1.1. Polynomial Order and Number of PCs

As described above, the spectral reconstruction of 40 combinations of different polynomial items (or orders) and the number of *PCs* (the principal components involved) was conducted; the averages of the evaluation results are illustrated in Figure 4. For simplicity, the training samples were selected from all 514 samples, and the number of samples in the training set was set to 128, i.e., approximately one-fourth of the total number. Thus, the ratio of the numbers of the training set and verification set was 1:3. Previous calculations have indicated that this sample ratio performs better. Table 1 lists the 40 parameter combinations of the number of *PCs* and items of polynomial extensions.

**Figure 5:**
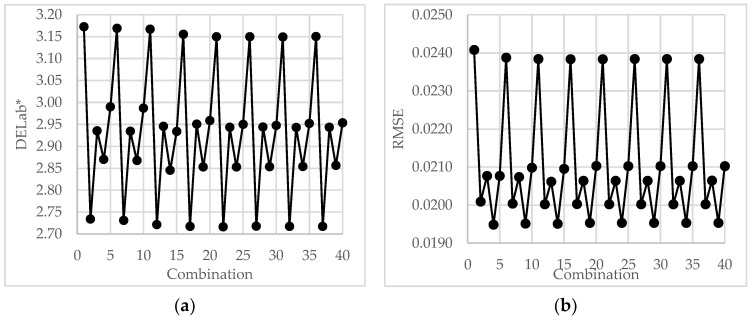
Global performance of combinations of different polynomial items and numbers of *PCs*. (**a**) Color errors in terms of DELab*, (**b**) Spectral errors in terms of *RMSE*.

Figure 5 illustrates the results. From Figure 5a, we can see that the combination 2,7,12,17,22,27,32,37 achieved the best group aligning in the lowest line. Looking at Table 2, we find that all the combinations of the best group have four polynomial items, in which the numbers are displayed in bold. That is to say, the *RGB* response of the camera extended by *first* order polynomial had the best performance in terms of ${∆E}_{{ab}^{*}}$. However, the performances of the *third* order (20 items) polynomial demonstrated superior results compared to the others in terms of *RMSE*, as shown in Figure 5b.

Based on the above results, the optimal parameter of a polynomial could be considered to be *1^st^-order*, namely, comprising four items. This is favorable to the evaluation index DELab*, although the *RMSE*s are trivially worse than those with *3^rd^ -order* (20 items). There are two reasons for this. Firstly, from Figure 5a and Table 1, we can see that the difference between the *first* and *third* order polynomials was less than 0.2 Lab* units, which was far less than the discrimination capabilities of human eyes [22]. Secondly, because the *1^st^-* and *3^rd^-orders* had 4 and 20 items respectively, the computational requirements of the *first order* algorithm were far less than those of the *third order*.

It should be mentioned that the DELab*s in this article (otherwise Specified) was calculated by the means of the median DELab* under each of the four illuminations (*A, D50*, *F02*, and *D65*). This was intended to provide the conditions for a robust assessment of the metameric color match determined by the precision of the spectral reconstruction rather than being limited in terms of color matching under specific illumination conditions [8].

#### 5.1.2. Size of Training Sets and Number of Iterations

**Figure 6:**
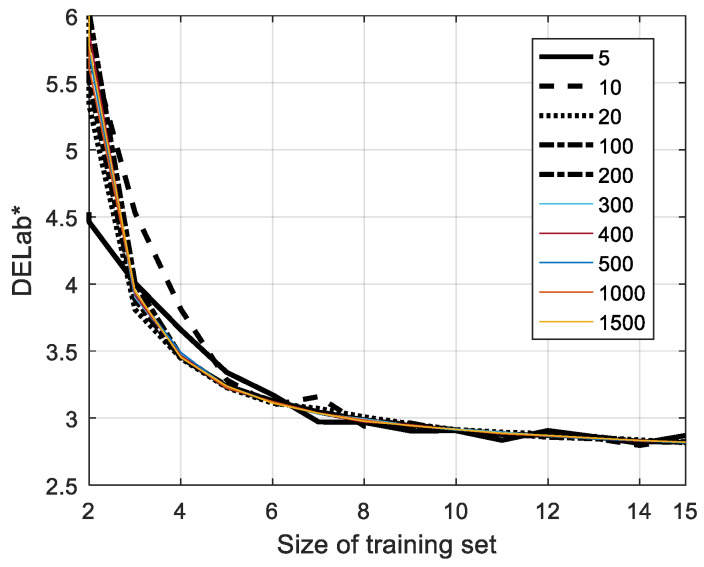
Stability of the proposed algorithm when varying the iteration number and the size of the training set.

Figure 6 demonstrates the stability of the *HPCAPR* var hybrid algorithm when varying the iteration number of random sampling and number of sampling subjects in the training set. Note that DElab*s is the average of the median results of the spectral reconstruction performance of the verification set when selecting a training set randomly for specific iteration numbers. In Figure 6, we can see that the results get worse when the size of the training set is less than eight subjects and the iteration number is less than 20. Otherwise, the results reveal a relatively high level of stability, regardless of the number of iterations, when the subjects and the iteration number are greater than 10 and 20. Therefore, we adopted safety parameters according to this protocol.

**Figure 7:**
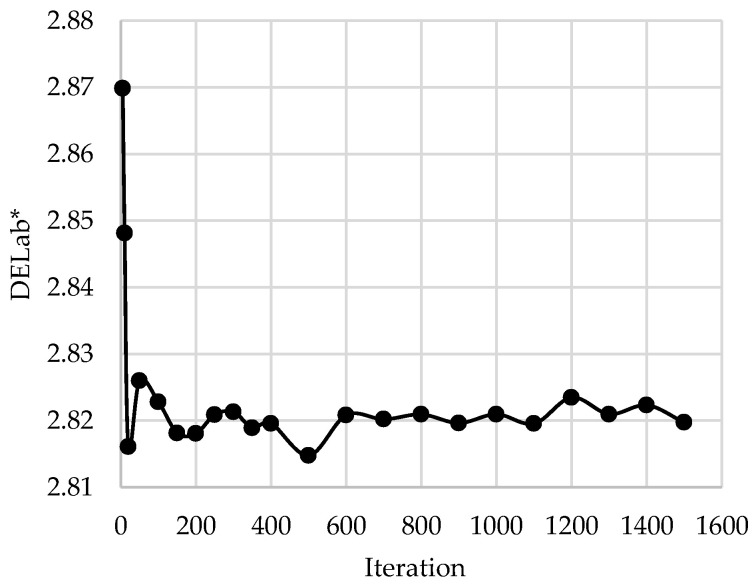
Stability of the algorithm when varying the iteration number of 10 random samples as the training set.

Figure 7 displays the results of the DELab* metrics of the skin spectral reconstruction by *1^st^-order* polynomial and the *3PCs* hybrid algorithm with 10 sampling subjects varying with the corresponding iteration numbers. As displayed in Figure 7, the performance rapidly improved with an iteration number larger than 20, although minor fluctuations remained. Surprisingly, under the minimum iteration number, i.e., 5, the evaluation value in terms of the color difference error DELab* was acceptable, i.e., 2.87 [22].

#### 5.1.3. Comparison with Separate PCA and the Polynomial Method

For a quantity comparison, Table 3 lists the evaluation results of *1^st^-order PR* (polynomial regression) and the *3PCs plus PCA* hybrid spectral reconstruction algorithm with the dataset with both ethnicities when the number of the training set was 40. The protocol was the same as described above, in which the data were the average of the median of the corresponding results. From Table 3, we can see that the *3PCs+1^st^-Order* algorithm achieved the best performance. That is to say, separate *PR* or *PCA* was worse than *PR plus PCA*, in that it decreased the ill condition of the transformation between the *RGB* space and the spectral reflectance space.

These results evidence the superiority of the proposed HPCAPR to any other previous method. This can be confirmed by comparing with Table 1 and Table 2, and the computational results listed in Table 3.

**Figure 8:**
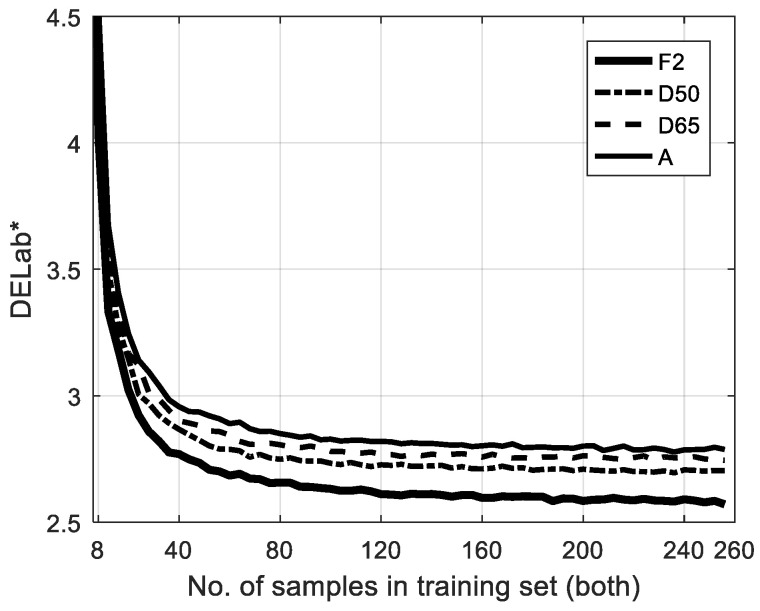
General performance of *3PCs plus First Order* algorithm when varying the size of training sets and the illumination.

The methods listed in Table 1 can be classified into two fundamental categories. *PR* [21], *PRPCA* [22], *PRPCAR* [25], and *HPCAPR* comprise the first type, in the sense that they do not only use *PR* but also the *PCA* method. In the second approach, the fundamental method is the *first-order* polynomial regression used in *RFOPR* [23] and *P2XYZ* [24]. From Table 3, we can see that the algorithm that adopted the *3PCs+First Order* combination attained the best performance with all of the 40 possible parameter combinations related in Table 2. Moreover, in contrast to previous authors that gave the results corresponding to several CIE standard illuminations, Figure 8 graphs the performance of the algorithm with the same *3PCs plus 1^st^-Order* algorithm configuration. We can see that the results are consistent with previous research, especially regarding the best performance under F2 illumination [22,25]. Therefore, the proposed *HPCAPR* framework should adopt this algorithm configuration, which has been shown to be the best choice, both in principle and by experimental computation.

In the first half of this paper, we confirmed that the performance of the hybrid of *PCA* and *RGB* polynomial algorithms with *3PCs plus 1^st^-Order PR* gives the best results among the existing methods listed in Table 1. We also determined the optimal parameters to be used for further investigation in the second half of this report. Our motivation was to further determine the optimal subspace of skin reflectance and clustering methods in order to further improve the spectral reconstruction performance, as outlined in Section 5.2.

### 5.2. Spectra Reconstruction in Clusters Classified in a Subordinate Color Space

#### 5.2.1. Protocol

For the sake of focusing on the subspace which will be explored in the following sections, we outline the following experimental protocols:

1. The iteration times were fixed at 300.
2. The size of the training set was 40 samples, selected randomly from a larger ensemble set containing 608 samples or a subset thereof; the verification set comprised the rest of the samples of the corresponding dataset or subset.
3. The CIE standard illuminant *D65* was adopted.
4. The evaluation index adopted DELAB* (${∆E}_{ab*}$) color difference, regardless of the highly correlated spectral error index *RMSE*, and the subindices included *mean*, *median*, *maximum*, *minimum*, and *standard deviation*.
5. Among the 300 iterations, the results of the best and worst groups in terms of mean ${∆E}_{ab*}$ are demonstrated.
6. Five groups of results are given considering random variations in the *k-means* algorithm.

#### 5.2.2. Spectral Reconstruction in the 2D La* Color Subspace

**Figure 9:**
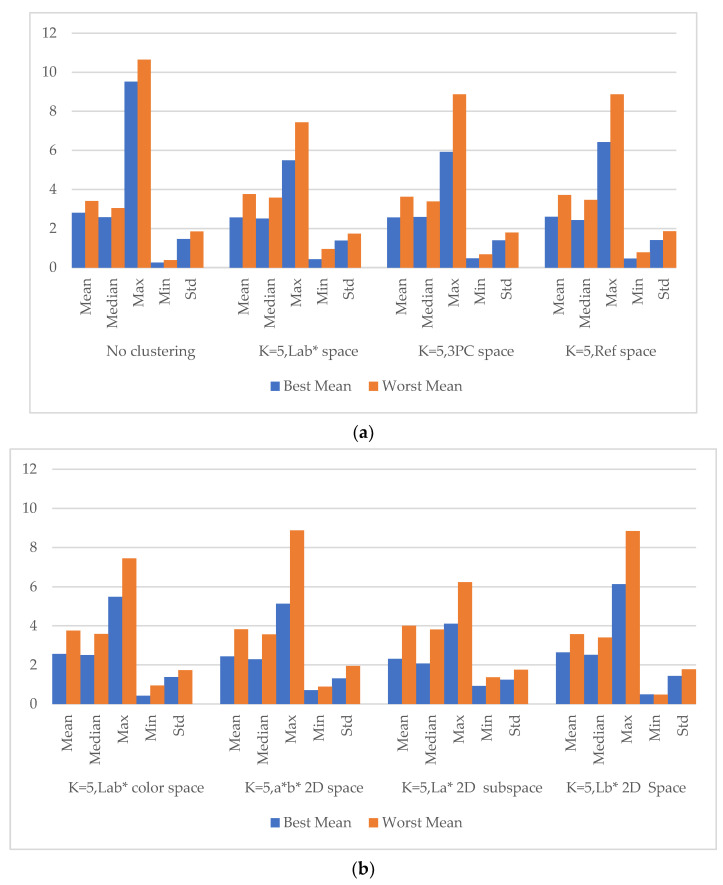
Performance of skin reflectance reconstruction for the proposed *HPCAPR* framework under various clustering strategies. (**a**) Clusters in the spaces with dimensions not less than three. (**b**) Clusters in CIELab* and its subordinate *2D* color spaces. Note that the vertical axis refers to DELab*(${∆E}_{ab*}$). Every index number has been averaged from five repetitions with 300 iterations each.

Figure 9 summarizes the performance of skin reflectance reconstruction for the proposed *HPCAPR* framework under various clustering strategies, in which the number of centroids *K* = 5. Note that *K* has the same meaning as *k* in Equation (21). In the computation process, we found that the value of *K* was constrained by the overall number of samples in the dataset, in which it was hard to gain high precision results if *K* was too small. In contrast, if it was too large, the algorithm collapsed due to the number of samples in the training set going beyond the number of all the samples in a cluster. From Figure 9a, we can see that pursuing subsets is an effective way to improve the precision of the spectral reconstruction in the clusters. The CIELab* color space outperformed the *3D 3PC* space (the space that spanned by the first three *PCs* of the reflectance of the dataset) and the 31 dimensional reflectance space. Figure 9b indicates that La* is the best corresponding *2D* color subspace.

#### 5.2.3. Clustering in the 2D La* Color Subspace by *Cosine Distance*

It is worth mentioning that the results in Figure 9 are clustered in the ‘*sqeucilidean*’ distance (Equation (22)), which is the default distance in many clustering applications. In this research, however, we found that the most appropriate distance was the ‘cosine’ distance, as described as Equation (24). Together with the ‘*cityblock*’ distance, the evaluation results are listed in Table 4. There, we can see that the median and maximum of the ‘*cosine*’ distance are better than those of the ‘*sqeuclidean*’ distance. The two main statistics are of the utmost significance for color reproduction. The median is clearer for the distribution than the mean, especially for a sparse distribution of bigger values. As an example, we can see that the median is smaller than the mean in Table 4. Therefore, we can safely conclude that the ‘cosine distance’ performs better.

### 5.3. Improvement of the Proposed HPCAPR Framework

As addressed above, the proposed *HPCAPR* framework for facial skin reflectance reconstruction comprises two main topics. One is the hybrid *3PCs plus 1^st^-order PR* algorithm, which reduces the ill condition of the transformation between the reflectance and the camera response; the other is the spectral reconstruction of clusters classified in the 2D La* color subspace. To the best of our knowledge, the present research is the first proposed method for skin spectra reconstruction via clustering in the 2D La* color subspace. Table 5 lists the results of the proposed *HPCAPR* (left columns) and those of similar algorithms without using the clustering technique. If we focus on the median and maximum errors, we can quantify the improvement. For the best cluster, we attained 25.2% and 57.1%; for the worst, the maximum error was reduced by 42.2%.

**Figure 10:**
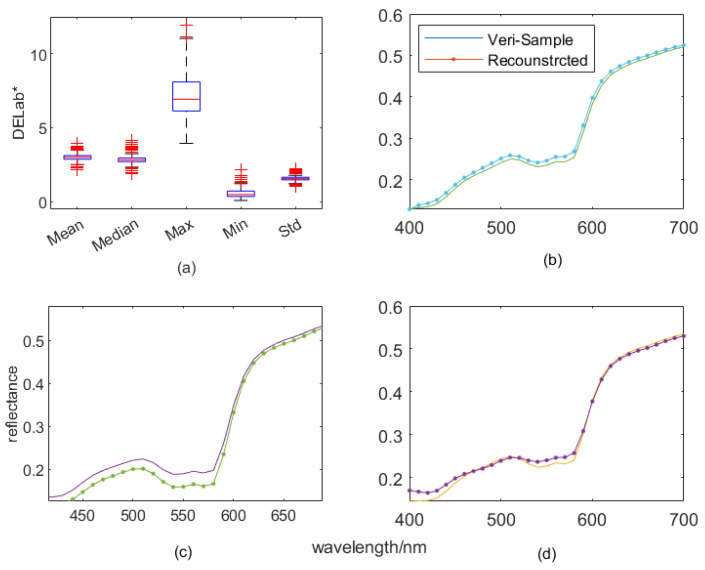
Evaluation index distribution for the best clusters, after 300 iterations and using three samples, of the reconstructed spectra curves. (**a**) Boxplot of evaluation index distributions. (**b**–**d**) Contrasts of the reconstructed reflectance spectra and corresponding measured counterparts in the verification set. Statistically, outliers are considered to be the most extreme data and are plotted individually using the red ‘+’ marker symbol in (**a**). Note that the labels of panels (**b**–**d**) use the same ‘wavelength’ *xlabel*s and ‘reflectance’ *ylabel*s; the colors of curves in panels (**b**–**d**) are random generated by the plotting tool.

It might be surprising to see the maximum figures reversed in the worst clusters indicated in the last line in Table 3; however, one might also notice that the sparse outliers in the dataset impact more on the figures in the clusters than in the ensemble dataset. This is a consequence of the average operation on the outliers (extreme data) not being favorable to the clusters with fewer samples. Figure 10a illustrates the distribution of the statistics. We can see in the boxplot that the outliers were impacted significantly by the means or medians. As demonstrated in Figure 6, more samples in the training set does not increase the operational precision; on this basis, we can also explain the impact of the outliers on the overall performance of the reflectance reconstruction system. That is to say, there is not a significant increase in performance, although the training set could have more samples in certain circumstances without using the clustering strategy. To intuitively demonstrate the performance of the proposed *HPCAPR*, Figure 10a–c displays three randomly selected spectra pairs of the reconstructed reflectance spectra and the corresponding measured counterparts in the verification set.

**Figure 11:**
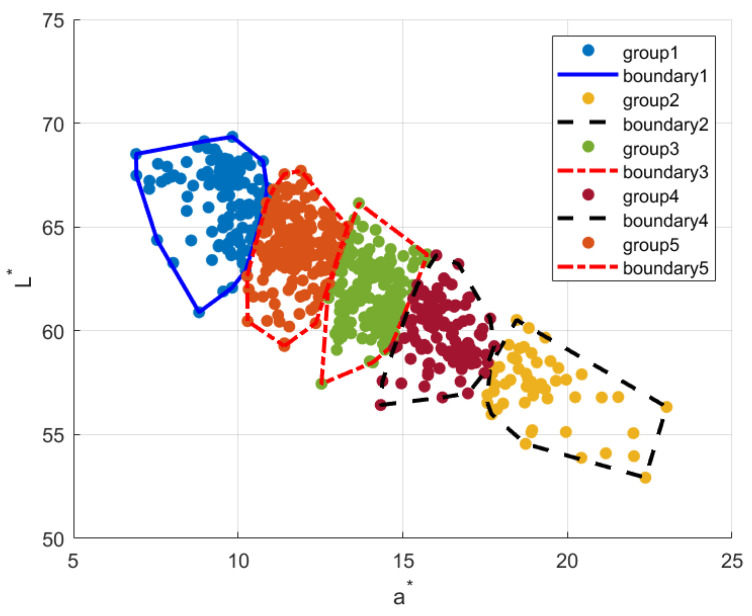
An example of the clusters after one *cosine distance* clustering operation.

For the sake of clarity, in Figure 11, we present a schematic of an example of the clusters of one clustering operation. Note that the cluster sequence is random because the initialization centroids were randomly selected [38]. Here, we can see the irregular distribution of the samples, especially for group5 and group1. The outliers in Figure 10a might be the sparsely distributed samples in such clusters. Finding the outliers and further reducing the maximum errors in skin reflectance reconstruction is beyond of the scope of this article.

We made preliminary comparisons in Section 5.1 and further investigations to find the optimal parameters for the proposed *HPCAPR* in Section 5.2 and Section 5.3. In Section 5.1, the *3PCs plus 1^st^-Order PR* algorithm was shown to conform to our expectations, revealing itself to be the best among existing algorithms. In Section 5.2 and Section 5.3, we applied the optimal space clustering in the 2D La* subordinate color space; the corresponding clustering distance was *cosine distance*. To the best our knowledge, this is the first algorithm which is able to perform reflectance reconstruction for human skin in the 2D La* color space.

## 6. Conclusions

We developed a practical *HPCAPR* framework for skin facial reflectance reconstruction from RGB images. Based on previous research and real human facial skin color datasets, we confirmed the effectiveness of the hybrid algorithm, which combines the *PCA* and *RGB PR* algorithms, in which the *3PCs plus first order PR* are the best parameter selection. Furthermore, we have demonstrated the superiority of subordinate 2D La* color subspace for facial skin spectra reconstruction from *RGB* images via a clustering technique. One limitation of the present research might be that the dataset contained images of subjects of only two ethnicities. However, race is an ambiguous term, and some Caucasians are darker than others [39]. Nonetheless, a dataset with more distributed samples could be investigated in the future.

## Figures and Tables

**Table 1:** A summary of spectral reflectance reconstruction methods from RGB color images.

Method	Ref.	Optimal Algorithm	*PCA* Source	Calibration Data
*PR*	F. H Imai, et al., 1996 [21]	*HDTV RGB* to *RGB* via *XYZ* by *PCA* method and *second* order polynomial regression	Skin reflectance dataset	108 reflectance spectra from 54 human faces
*PRPCA*	K. Xiao et al., 2016 [22]	Direct *RGB* polynomial regression to reflectance spectra via *PCA* method	Skin reflectance spectra dataset	Spectra matching silicon skin color chart
*RFOPR*	R. He et al., 2021 [23]	Raw *RGB* to reflectance spectra by *first* order polynomial regression	Not applicable	200 pieces of skin data collected using five facial locations on 40 human faces.
*P2XYZ*	R. He et al., 2021 [24]	*RGB* to *XYZ* via *first* order polynomial regression	Not applicable	facial skin data from 60 human faces
*PRPCAR*	L. Ma et al., 2021 [25]	*RGB* to reflectance spectra via *second* order polynomial regression plus *3PCs* with regulated denoise item	4392 pieces of data from a 482 subject database; different from the silicon skin dataset	90 pieces of skin data from a silicon skin database
*HPCAPR*	Proposed in this article	*RGB* to reflectance spectra by *first* order polynomial regression plus *3PCs* with modifying subset training	Skin reflectance selected uniformly from subsets	40 pieces of skin data uniformly selected from a *k-means* subset in the La* 2D color subspace

**Table 2:** Lists of the 40 parameter combinations of the number of PCs and items of polynomial extension.

Combination	1	2	3	4	5	6	7	8	9	10	11	12	13	14	15	16	17	18	19	20
Items	3	4	10	20	35	3	4	10	20	35	3	4	10	20	35	3	4	10	20	35
PCs	3	3	3	3	3	4	4	4	4	4	5	5	5	5	5	6	6	6	6	6
Combination	21	22	23	24	25	26	27	28	29	30	31	32	33	34	35	36	37	38	39	40
Items	3	4	10	20	35	3	4	10	20	35	3	4	10	20	35	3	4	10	20	35
PCs	7	7	7	7	7	8	8	8	8	8	9	9	9	9	9	10	10	10	10	10

**Table 3:** Performance with and without the combination of *PCA* and *PR*.

	*3PCs+1^st^-Order*	*1^st^-Order*	*3PCs*	*RGB*
DE Lab*	2.87	2.91	3.17	3.42
*RMSE*	0.0218	0.0221	0.241	0.0273

**Table 4:** Performance in terms of ${∆E}_{ab*}$ of different distances for the clustering algorithm in the 2D La* color subspace ^1^.

	Mean	Median	Max	Min	Std
	*K = 5*,’*sqeuclidean*’				
Best mean	2.31	2.06	4.10	0.92	1.24
Worst mean	4.00	3.81	6.23	1.36	1.75
	*K = 5*,’*cityblock*’				
Best mean	2.49	2.39	4.96	0.51	1.34
Worst mean	3.83	3.63	9.41	0.83	2.02
	*K = 5*,’*cosine*’				
Best mean	2.16	1.93	4.04	0.95	1.26
Worst mean	4.03	3.80	6.16	1.25	1.72

^1^ The best and worst means are the averages of five best- and five worst-performing clusters, respectively, as determined by the average of color differences ${∆E}_{ab*}$ of all the reflectance spectra in the verification set and the corresponding reconstructed counterparts in the cluster.

**Table 5:** Performance of clusters in the 2D La* color subspace, *Cosine* distance and five clusters ^1^.

	Cluster in La* Space					No Cluster				
Cluster	Mean	Median	Max	Min	Std	Mean	Median	Max	Min	Std
Best 1	2.12	1.93	4.01	0.99	1.24	2.8	2.56	9.41	0.26	1.43
Best 2	2.12	1.93	4.01	0.99	1.24	2.81	2.59	9.87	0.12	1.49
Best 3	2.12	1.93	4.01	0.99	1.24	2.8	2.57	9.41	0.38	1.5
Best 4	2.32	1.93	4.15	0.77	1.33	2.83	2.57	9.6	0.4	1.48
Best 5	2.12	1.93	4.01	0.99	1.24	2.8	2.6	9.28	0.11	1.43
Best Mean	2.16	1.93	4.04	0.95	1.26	2.81	2.58	9.51	0.25	1.47
worst 1	4.10	3.83	6.08	1.23	1.64	3.29	3.01	10.74	0.67	1.65
worst 2	4.10	3.83	6.08	1.23	1.64	3.43	3.05	9.44	0.28	1.9
Worst 3	4.10	3.83	6.08	1.23	1.64	3.37	3.09	10.31	0.43	1.74
Worst 4	4.10	3.83	6.08	1.23	1.64	3.45	3.04	10.26	0.32	1.89
Worst 5	3.76	3.68	6.48	1.32	2.05	3.5	3.01	12.49	0.2	2.05
Worst Mean	4.03	3.80	6.16	1.25	1.72	3.41	3.04	10.65	0.38	1.85

^1^ The left columns are from clusters classified using the *k-means* algorithm with ‘*Cosine*’ distance and five centroids.

## Data Availability

Not applicable.

## References

[1] Wang J., Yu X., Liu Q., Yang Z. (2019). Research on key technologies of intelligent transportation based on image recognition and anti-fatigue driving. EURASIP J. Image Video Process..

[2] Kim G., Ko H. (2018). A practical approach to Physically-Based reproduction of diffusive cosmetics. Comput. Graph. Forum.

[3] Li C., Zhou K., Wu H., Lin S. (2019). Physically-based simulation of cosmetics via intrinsic image decomposition with facial priors. IEEE Trans. Pattern Anal. Mach. Intell..

[4] Baranoski G.V., Chen T.F., Krishnaswamy A., Querleux B. (2014). Multilayer Modeling of Skin Color and Translucency. Computational Biophysics of the Skin.

[5] Widdowson D.C., Moore J.C., Wright P.A., Shakespeare P.G. (2010). Determination of the effects of blood depth in the dermis on skin colour in a novel skin phantom using digital imaging. Lasers Med. Sci..

[6] Guttman C. (2004). Cutaneous disease in skin of color surfaces as new vital field of study. Dermatol. Times.

[7] Sohaib A., Amano K., Xiao K., Yates J.M., Whitford C., Wuerger S.M. (2018). Colour quality of facial prostheses in additive manufacturing. Int. J. Adv. Manuf. Technol..

[8] Wyszecki G., Stiles W. (1982). Color Science: Concepts and Methods, Quantitative Data and Formulae.

[9] Yudovsky D., Pilon L. (2010). Rapid and accurate estimation of blood saturation, melanin content, and epidermis thickness from spectral diffuse reflectance. Appl. Opt..

[10] Anderson R.R., Parrish J.A. (1981). The optics of human skin. J. Investig. Dermatol..

[11] Van Gemert M.J., Jacques S.L., Sterenborg H.J., Star W.M. (1989). Skin optics. IEEE Trans. Biomed. Eng..

[12] Bashkatov A.N., Genina E.A., Tuchin V.V. (2011). Optical properties of skin, subcutaneous, and muscle tissues: A review. J. Innov. Opt. Health Sci..

[13] Fullerton A., Fischer T., Lahti A., Wilhelm K., Takiwaki H., Serup J. (1996). Guidelines for measurement skin colour and erythema, A report from the Standardization Group of the European Society of Contact Dermatitis. Contact Dermat..

[14] Piérard G.E. (1998). EEMCO guidance for the assessment of skin colour. J. Eur. Acad. Dermatol. Venereol. JEADV.

[15] Wang Y., Luo M.R., Wang M., Xiao K., Pointer M.R. (2017). Spectrophotometric measurement of human skin colour. Color Res. Appl..

[16] Wang M., Xiao K., Luo M.R., Pointer M.R., Cheung V., Wuerger S.M. (2018). An investigation into the variability of skin colour measurements. Color Res. Appl..

[17] Nkengne A., Robic J., Seroul P., Gueheunneux S., Jomier M., Vie K. (2018). SpectraCam^®^: A new polarized hyperspectral imaging system for repeatable and reproducible in vivo skin quantification of melanin, total hemoglobin, and oxygen saturation. Ski. Res. Technol..

[18] Gevaux L., Adnet C., Séroul P., Clerc R., Trémeau A., Perrot J.L., Hébert M. (2018). Three-dimensional hyperspectral imaging: A new method for human face acquisition. Electron. Imaging.

[19] Jacques S.L. (2009). Spectral imaging and analysis to yield tissue optical properties. J. Innov. Opt. Health Sci..

[20] Nishidate I., Maeda T., Niizeki K., Aizu Y. (2013). Estimation of melanin and hemoglobin using spectral reflectance images reconstructed from a digital rgb image by the wiener estimation method. Sensors.

[21] Imai F.H., Tsumura N., Haneishi H., Miyake Y. (1996). Principal component analysis of skin color and its application to colorimetric color reproduction on CRT display and hardcopy. J. Imaging Sci. Technol..

[22] Xiao K., Zhu Y., Li C., Connah D., Yates J.M., Wuerger S. (2016). Improved method for skin reflectance reconstruction from camera images. Opt. Express.

[23] He R., Xiao K., Pointer M.R., Bressler Y., Liu Z., Lu Y. (2021). Development of an image-based measurement system for human facial skin colour. Color Res. Appl..

[24] He R., Xiao K., Pointer M., Bressler Y., Liu Z., Lu Y. (2021). A novel camera color characterization model for the color measurement of human skin. Electron. Imaging.

[25] Ma L., Zhu Y. Skin spectral reconstruction in multispectral imaging. Image and Graphics Technologies and Applications. Proceedings of the 16th Chinese Conference on lmage and Graphics Technologies.

[26] Chong H.Y., Gortler S.J., Zickler T.E. The von Kries Hypothesis and a Basis for Color Constancy. Proceedings of the 2007 IEEE 11th International Conference on Computer Vision.

[27] Abdi H., Williams L.J. (2010). Principal component analysis. Wiley Interdiscip. Rev. Comput. Stat..

[28] Zhang X., Xu H. (2008). Reconstructing spectral reflectance by dividing spectral space and extending the principal components in principal component analysis. J. Opt. Soc. America. A Opt. Image Sci. Vis..

[29] Cheng C., Schneeweiß H. (1998). Polynomial regression with errors in the variables. J. R. Stat. Soc. Ser. B Stat. Methodol..

[30] Liu Z., Xiao K., Pointer M.R., Liu Q., Li C., He R., Xie X. (2021). Spectral Reconstruction Using an Iteratively Reweighted Regulated Model from Two Illumination Camera Responses. Sensors.

[31] Li S. (2018). Filter selection for optimizing the spectral sensitivity of broadband multispectral cameras based on maximum linear independence. Sensors.

[32] Cao B., Liao N., Cheng H. (2017). Spectral reflectance reconstruction from RGB images based on weighting smaller color difference group. Color Res. Appl..

[33] Kamimura K., Tsumura N., Nakaguchi T., Miyake Y. Evaluation and analysis for spectral reflectance imaging of human skin. Proceedings of the Color Imaging X: Process. Hardcopy Applications.

[34] Shen H., Xin J.H. (2007). Estimation of spectral reflectance of object surfaces with the consideration of perceptual color space. Opt. Lett..

[35] Liang J., Zhu Q., Liu Q., Xiao K. (2022). Optimal selection of representative samples for efficient digital camera-based spectra recovery. Color Res. Appl..

[36] Li S.-X. (2021). Superiority of optimal broadband filter sets under lower noise levels in multispectral color imaging. Color Res. Appl..

[37] Arthur D., Vassilvitskii S. (2007). k-means++: The advantages of careful seeding. SODA ‘07. Proceedings of the Eighteenth Annual ACM-SIAM Symposium on Discrete Algorithms.

[38] Weyrich T., Matusik W., Pfister H., Bickel B., Donner C., Tu C., McAndless J., Lee J., Ngan A., Jensen H.W. (2006). Analysis of human faces using a measurement-based skin reflectance model. ACM Trans. Graph..

[39] Fitzpatrick T.B. (1988). The Validity and Practicality of Sun-Reactive Skin Types I Through VI. Arch. Dermatol..

